# Detailed analysis of surgically treated hand trauma patients in a regional German trauma centre

**DOI:** 10.1371/journal.pone.0283388

**Published:** 2023-03-24

**Authors:** Mechthild Gerken, Maximilian Michael Saller, Ben Ockert, Wolfgang Böcker, Elias Volkmer

**Affiliations:** 1 Department of Orthopaedics and Trauma Surgery, Musculoskeletal University Centre Munich, University Hospital, Ludwig-Maximillians-University, Munich, Germany; 2 Department of Hand Surgery, Helios Klinikum München West, Munich, Germany; BG Trauma Center Ludwigshafen, GERMANY

## Abstract

Hand and forearm injuries are the most frequent reason for consultations in German emergency departments. Therefore, full recovery has a high social and economic relevance. In this study, data on surgically treated hand injuries in a regional German trauma centre between 01.01.2019 and 31.01.2021 were collected using the new German HandTraumaRegister of the German Society for Hand Surgery. These data were retrospectively analysed and correlated with mobility data of the Bavarian population, the 7-day incidence of Covid-19 infections in Germany and the number of elective hand surgeries. We found that a fall from standing height with consecutive distal radius fracture was the most common injury in women, whereas mechanism of injury and diagnosis were more diverse in men. The populations’ mobility correlated well with the number of accidents, which in turn was reciprocal to the 7-day-incidence of Covid-19 infections. The number of elective hand surgeries expectedly dropped significantly during the state-imposed lockdowns. Knowing that mainly young men and elderly women suffer from hand injuries, tailored prevention measures may be elaborated. In order to reduce socioeconomic burden, care for hand injuries and elective hand surgeries must be guaranteed according to the frequency of their occurrence.

## Introduction

Hand injuries are the most frequent reason for consultations in German general emergency departments [1]. Hand and forearm injuries also represent the most frequent reason for occupational accidents (41%) and hence cause the majority of new accident pensions [2]. According to the Federal Institute for Occupational Safety and Health in Germany, injuries, poisonings and occupational accidents accounted for a total of 9.3 billion euros in lost production costs in 2019 [3]. In addition, industry assumed 12.4 billion euros in apportionment costs for the employers’ liability insurance association in 2019 [4]. Therefore, an adequate and timely surgical treatment of hand injuries is not only crucial for regaining patients’ quality of life, but also of outstanding socioeconomic importance.

However, there is little data on the epidemiology of severe hand injuries, which makes it difficult to develop prevention strategies and to maintain appropriate treatment modalities in emergency centres as well as to plan staffing for surgical treatment capacity in hospitals. The aim of our study was therefore to analyse relevant hand surgery emergency patients in detail in order to provide data for prevention and planning of appropriate treatment using the epidemiological data of the new German”HandTraumaRegister German Society for Hand Surgery” (*HandTraumaRegister Deutsche Gesellschaft für Handchirurgie*, HTR DGH). From 01.01.2019 to 31.01.2021, we investigated all hand trauma cases that required surgery in our regional trauma centre. The analysis of the number of surgically treated elective patients served as a comparative value to the surgically treated hand trauma patients.

As the Covid-19 pandemic was ongoing during the evaluation period, pandemic related factors such as the populations`mobility and activity level of the population during lockdowns were documented. This coincidence provided the rare opportunity to assess the impact of important parameters such as a massively reduced mobility and activity of the entire population on the prevalence of hand trauma. The present study thus unveiled peculiarities of surgically treated hand trauma patients in terms of case numbers, injury patterns and causes of accidents in comparison to elective patients that may be utilized to develop prevention strategies and to plan and provide appropriate emergency care structures.

## Methods

### Study design

For this monocentric study performed at a regional trauma centre in Germany, data of surgically treated hand trauma patients from 01.01.2019 to 31.01.2021 were evaluated. During this period, a significant change occurred in population activity due to the corona virus pandemic. Hand trauma patients from the pre-pandemic year 2019 were considered as the ‘control group’. Likewise, the data of elective hand surgery patients from 04.07.2019 to 31.01.2021 were analysed. The hospital “Helios Klinikum München West”, a regional trauma centre, provides care for the entire western part of Munich, including the surrounding area. Due to the large catchment area with many residential areas of all population strata and industry, this monocentric study represents the broad spectrum of possible hand injuries in a society.

The inclusion criteria of the HTR DGH were used for data collection. Patient cases of this hospital have been entered in the HTR DGH since the 01.01.2020.

### Data collection

The inclusion criterion for the HTR DGH is an injury in the area of the distal third of the forearm or hand that was surgically treated within 14 days of the traumatic event and recorded with the help of the “German procedure classification” (*Operationen- und Prozedurenschlüssel*, OPS) of the “German Institute for Medical Documentation and Information” (*Deutsches Institut für Medizinische Dokumentation und Information*, DIMDI) [5]. A conservatively treated injury is an exclusion criterion for the HTR DGH. Furthermore, the time of surgery of elective hand surgery patients were collected.

**Fig 1:**
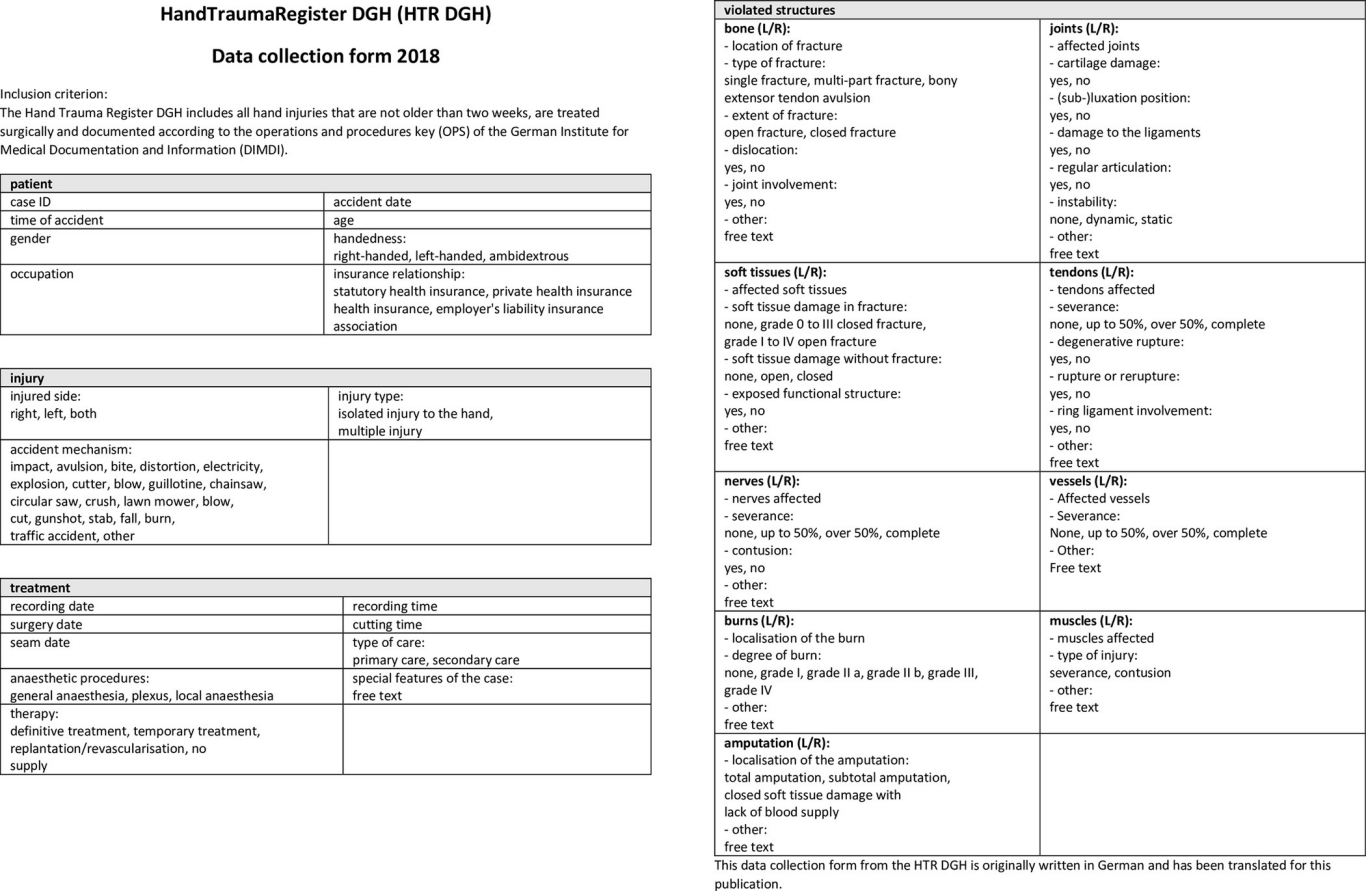
Data collection form of the HTR DGH [5].

In our study, gender, age, cause of accident, diagnosis, date of accident and date of operation were documented (Fig 1).

Each patient case was evaluated once. Patients were grouped into age decades. Due to the absence of a paediatric surgery department in the hospital, the age decade 0–10 years was excluded. The diagnoses were classified into the following categories: Distal radius fracture; metacarpal fracture; finger fracture; amputation; carpal fracture; tendon, nerve, vascular injury and “other injuries”. The causes of accidents were documented in the following categories: falls from standing height; sporting accidents; fist punches; occupational accidents; stabbings, cuttings, circular saw injuries; distortions; impact traumas and “other causes of injuries”.

All patients registered in the German HTR DGH have signed an informed consent to anonymised scientific evaluation of their data. The analysis of the data was approved by the hospital’s internal research department and the ethics committee of the Ludwig-Maximilians-University, Munich (project number: 21–1052).

### Data extraction and analysis

Data were extracted from the patient hospital system” CompuGroupMedical” (CGM) Medico (Koblenz, Germany) from 01.01.-03.07.2019 and the patient hospital system “Systemanalyse Programmentwicklung” (SAP) (Walldorf, Germany) from 04.07.2019. The data were entered into the HTR DGH online. Data analysis was performed descriptively and exploratively using the data analysis programme R/RStudio.

For the daily patient cases of the year 2020 and January 2021, the hand trauma patients on the one hand and the elective patients on the other hand, the sliding average over seven days was calculated and presented as a line chart. The comparative period 2019 of the hand trauma case numbers was presented as the mean value of the individual time periods.

The 7-day-incidence of Covid-19 patients in Germany was provided by the Robert Koch Institute [6]. The 7-day-incidence in Germany, as far as the data are available, corresponds to the 7-day-incidence of Bavaria. Since the 7-day-incidence in Germany was made available to the public at an earlier point in time, these data were used. The population size in Germany as of 31.12.2019 and the activity level of society were both provided by the Federal Statistical Office [7]. The activity level was calculated using the analysis of mobile phone data from the mobile phone provider Telefónica in Bavaria 2020 and January 2021 compared to 2019 [8] and presented as a moving average over seven days.

The number of hand trauma surgeries per day and the number of elective surgeries per surgery day were plotted using box-whisker plots and the mean value. The Wilcoxon test was used to determine the p-value in a two-sided test, the significance level was set at *p*<0.05. The relative ratios of diagnoses, age decades and causes of accidents of hand traumatology patients were shown in bar diagrams for the overall period as well as for the individual time periods.

### Time periods

The data were cumulatively divided into the different time periods of the pandemic, according to the contact restrictions imposed by the Bavarian government: *Pre-pandemic phase*, *first lockdown* [9], *intermediate phase* [10], *lockdown light* [11] and *second lockdown* [12] (Table 1).

**Table 1:** Time periods of the Covid-19 pandemic.

designation of the time period	period of time
***pre-pandemic phase* (before the first lockdown in 2020)**	01.01.2019–16.03.2020
***first lockdown* (2020) [9]**	17.03.2020–05.05.2020
***intermediate phase* (after the first lockdown 2020) [10]**	06.05.2020–01.11.2020
***lockdown light* (2020*)* [11]**	02.11.2020–15.12.2020
***second lockdown* 2020/2021 [12]**	16.12.2020–31.01.2021

In the course of the pandemic, the period of the *first lockdown* was defined as the first wave, the *lockdown light* and *second lockdown* as the second wave.

In two time periods, from 01.04.-07.04.2020 and from 18.12.-31.12.2020, the hospital was deregistered from the interdisciplinary care record (*Interdisziplinärer Versorgungsnachweis*, IVENA eHealth) due to high Covid-19 infection numbers. IVENA eHealth is an information system for the transmission of free capacities of hospitals to the control centre of the emergency service in order to distribute patients to the emergency rooms according to the free resources. Therefore, no new patients were brought to this hospital by ambulance during this period. Since hand trauma patients also came to the hospital on foot, the IVENA eHealth deregistration had only a minor impact on the case numbers.

## Results

A total of 1218 patient cases (617 elective and 601 trauma cases) were recorded. The inclusion rate in the HTR DGH was determined for the year 2020 and January 2021, according to the start of documentation in the HTR DGH from 01.01.2020 (Table 2).

**Table 2:** General case numbers of traumatological and elective patients per calendar year and inclusion rate in the HTR DGH.

Calendar year	Number of hand trauma patients	Inclusion rate in the HTR DGH	Number of elective patients
**2019**	276	-	195 (from 04.07.2019)
**2020**	295	78,31%	411
**January 2021**	30	73,33%	11

### The most frequent diagnosis in surgically treated women within the hand surgery department is the distal radius fracture, in men it is the metacarpal fracture

**Fig 2:**
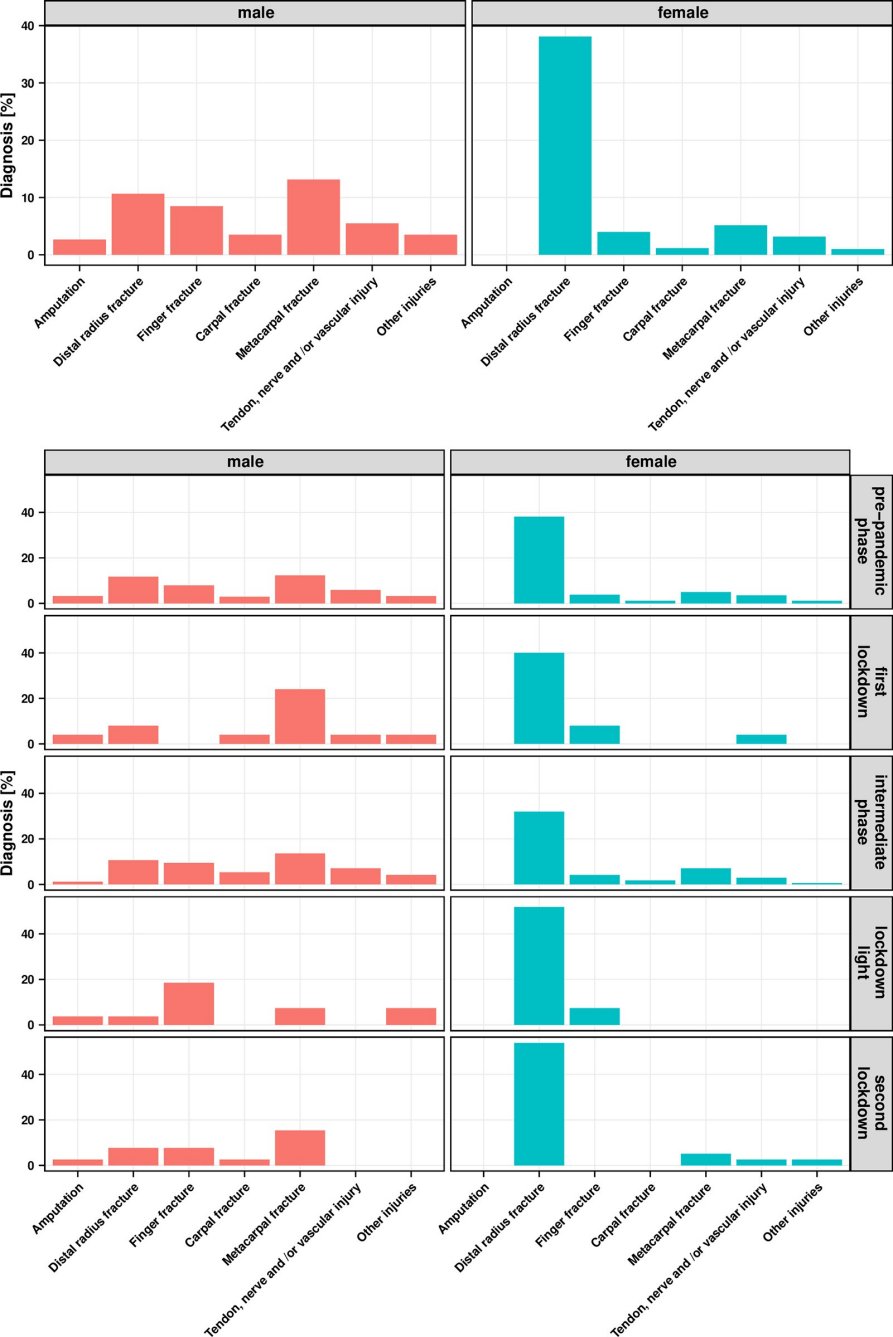
Relative proportion of diagnoses versus gender (male and female = 100%). A) in the entire observation period from 01.01.2019–31.01.2021. B) per pandemic interval.

The analysis of all hand trauma cases by diagnosis and gender showed that almost 40% of all injuries in female patients involved a fracture of the distal radius. In contrast, a broader spectrum of diagnoses was found in male patients. At almost 15%, the metacarpal fracture was the most frequent injury here, followed by the distal radius fracture and finger fracture (Fig 2A). During the lockdown phases, we found a decrease of all diagnoses except for the distal radius fracture. The distal radius fracture was the most common diagnosis in female patients at any time during the pandemic. Among the men, it was noticeable that the metacarpal fracture occurred more frequently in the *first lockdown* phase, while finger fractures were then completely absent. However, this ratio was reversed again in the *lockdown light* phase. Otherwise, the distribution of diagnoses among male patients changed little over the pandemic phases (Fig 2B).

### Falls from standing height are the most frequent cause of accidents in females

**Fig 3:**
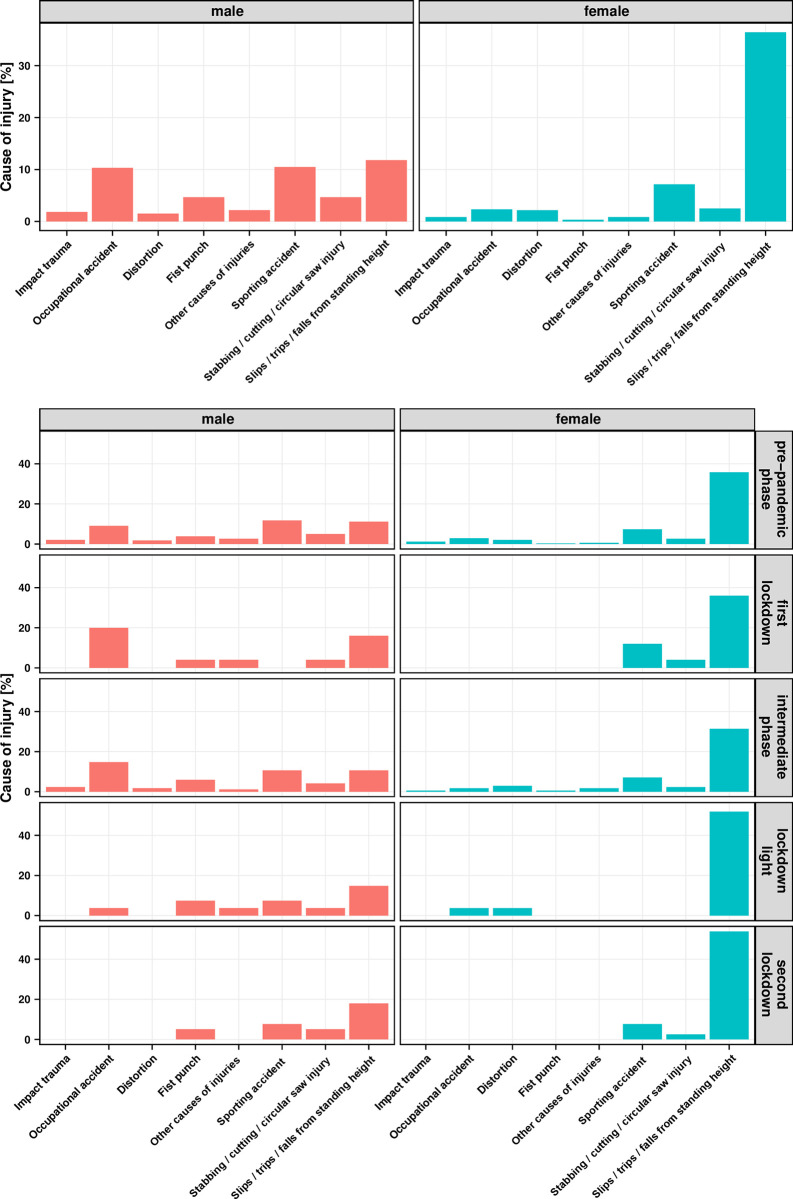
Relative proportion of causes of accidents versus gender (male and female = 100%). A) in the entire observation period from 01.01.2019–31.01.2021. B) per pandemic interval.

In order to examine the risk factors of hand injuries in more detail, the causes of accidents, including gender differences, were examined. In the overall period, falls from standing height were the most frequent cause of accidents among female patients, accounting for almost 40%. Among the men, the distribution of causes was broader. With more than 10% each, they included the falls from standing height, sporting accidents and occupational accidents (Fig 3A).

Detailed analysis of the causes of accidents during the pandemic year showed that many types of injuries were not present in the female patients during the lockdown phases. The falls from standing height in turn persisted as the main cause of accidents. Interestingly, however, during the *first lockdown*, only female patients in our collective suffered sporting accidents. Men, on the other hand, had more accidents at work during the *first lockdown*, but no sporting accidents (Fig 3B).

### The age structure of men and women with hand injuries is inverse

**Fig 4:**
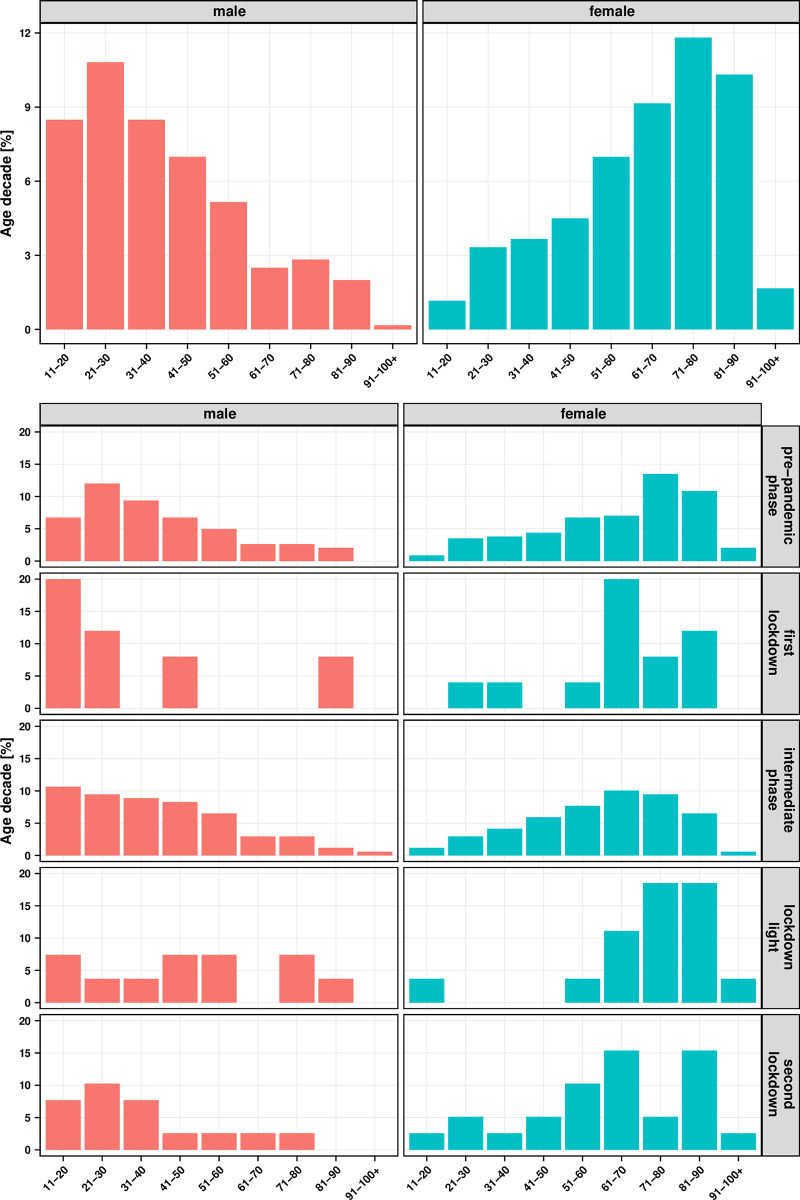
Relative proportion of injuries per age decade (male and female = 100%). A) in the entire observation period from 01.01.2019–31.01.20221. B) per pandemic interval.

The breakdown of the age structure of male and female patients provided important information about the age of the typical patient clientele in hand traumatology. While the frequency of accidents in male patients decreased sharply with age, the frequency of accidents in female patients was increasing tremendously (Fig 4A). In addition, both in the *first lockdown* and in the *intermediate phase*, a shift of the age distribution by one decade to the left was observed for both sexes, which means that the average age of the hand injured patients decreased (Fig 4B).

### Dynamics of hand trauma case numbers follow the mobility of the population during the pandemic

**Fig 5:**
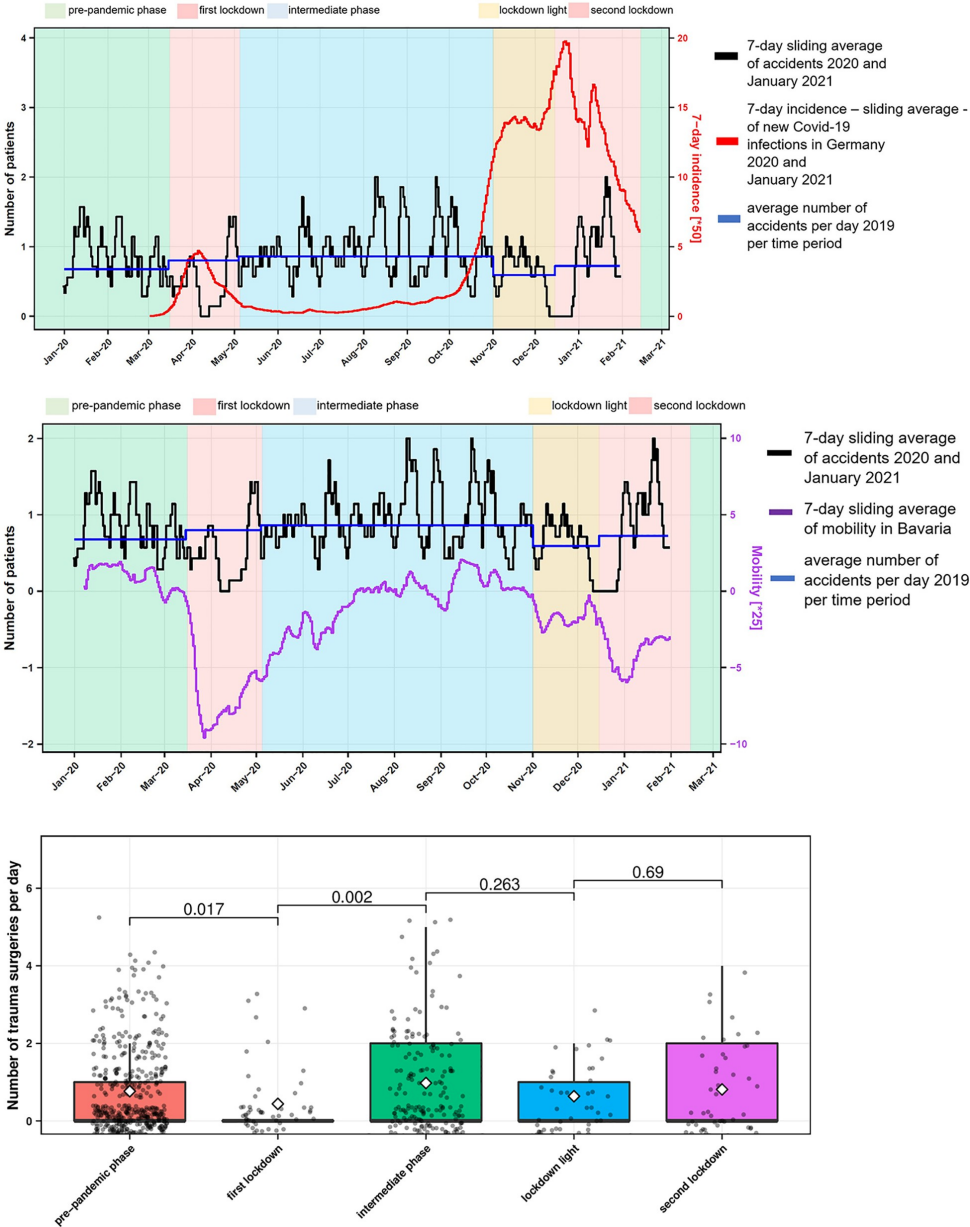
Number of hand trauma patient cases. A) accidents per day in 2020 and January 2021 versus new Covid-19 infections in Germany per day and accidents per day 2019 per time period. B) accidents per day 2020 and January 2021 versus mobility in Bavaria as well as accidents per day 2019 per time period. C) number of hand trauma operations per day per pandemic interval and p-value.

The correlation of hand trauma cases with the population mobility and the 7-day-incidence of new Covid-19 infections showed that during the *first lockdown*, hand accidents and mobility decreased dramatically, while the 7-day-incidence of Covid-19 infected individuals skyrocketed. At the end of the *first lockdown*, the number of people infected with Covid-19 declined and hence mobility and incidence of hand injuries increased again until, as of August 2020, they were significantly higher than in the same period of the previous year 2019. Shortly before the start of the *lockdown light*, case numbers of Covid-19 increased, while at the same time the incidence of accidents and the general mobility decreased. In the *second lockdown*, the number of people infected with Covid-19 decreased, but the frequency of accidents and general mobility increased. The average number of hand accidents from 2019 was used for comparison (Fig 5A and 5B).

While the number of hand trauma operations significantly dropped from an average of 0.8 (standard error of the mean, SEM 0.05) patients per day in the *pre-pandemic phase* to 0.4 (SEM 0.13) patients per day in the *first lockdown* (*p* = 0.02), it significantly increased to 1.0 (SEM 0.1) traumatological operations per day in the *intermediate phase* (*p* = 0.002). The mean value dropped to 0.64 (SEM 0.12) in the *lockdown light* and rose again to 0.8 (SEM 0.16) traumatological operations per day in the *second lockdown* (Fig 5C).

### Strong fluctuation of elective case numbers according to lockdown prescriptions

**Fig 6:**
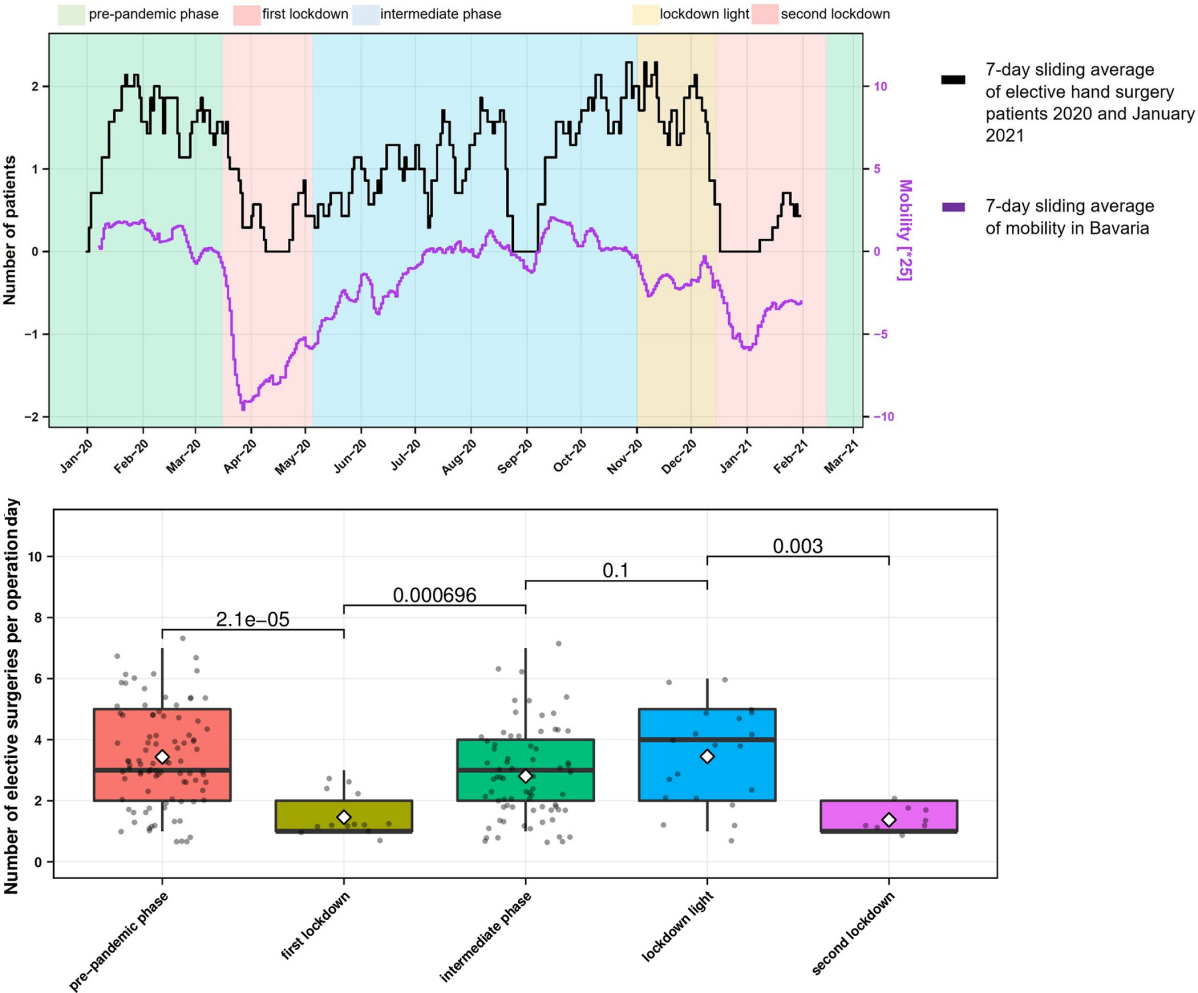
Number of elective case numbers. A) elective patients per day 2020 and January 2021 versus mobility in Bavaria B) number of elective surgeries per day per pandemic interval and p-value.

Over time, a correlation between elective surgery numbers and the mobility behaviour of the population was shown (Fig 6A). The number of elective surgeries per surgery day decreased significantly from the *pre-pandemic phase* (mean 3.44; SEM 0.17) to the *first lockdown* (mean 1.46; SEM 0.22) (*p*<0.001). In the *intermediate phase* (mean 2.81; SEM 0.17), the number of elective surgeries increased significantly (*p*<0.001) until it peaked at the beginning of the *lockdown light* (mean 3.45; SEM 0.37). At the *second lockdown* (mean 1.38; SEM 0.18), the numbers decreased significantly again (*p* = 0.003) (Fig 6B).

## Discussion

Although hand injuries are numerically the most common type of injury in German emergency departments [1], reliable data on the epidemiology of hand injuries is scarce. For this reason, our study examined the risk factors and types of injuries as well as injury mechanisms of surgically treated patients with hand injuries. The prevailing pandemic situation in 2020 and January 2021 provided the unique opportunity to shed light on the influence of factors such as mobility and activity on the incidence of hand injuries.

### Distribution structure of injuries in the course of the pandemic

The distribution of diagnoses in a gender comparison showed that 40% of all women in this study suffered a distal radius fracture. Numerous studies investigated the incidence factors of distal radius fractures and confirmed this distribution of hand trauma patients especially for high age decades [13]. One of the main reasons for this is probably the population structure: 66.1% of the over 85-year-olds are female in Germany [14]. In addition, osteoporosis, which has a clear gender-specificity, certainly plays an important role [15]. Thus, the same slip, trip or fall from standing height may induce an injury in a woman while it does not in a man. The fact that the incidence of the distal radius fractures did not decrease during the lockdown shows that domestic accidents are a frequent cause, which in turn is an important starting point for prevention campaigns. A women-focused campaign may draw attention to this issue and help to reduce the number of fractures. Regardless of the pandemic situation, the spectrum of diagnoses was more widely distributed among male patients in our study. This phenomenon is also indicated by a study by Court-Brown et al. which showed that finger fractures and metacarpal fractures were diagnosed more frequently in men than in women [16]. The increase in metacarpal fractures in men during the *first lockdown* seemed to be due to an increased number of so-called boxer’s fractures. Whether it can be explained with the fact that men reacted with increased aggressiveness to the lockdown measures is probably beyond the informative value of the present data set, but it would at least be conceivable.

### Distribution of causes of accidents in the course of the pandemic

The causes of hand injuries are manifold. It is interesting that women reported falls from standing height as the most frequent cause of injury. It is known that both vitamin D deficiency [17] and increasing age [18] cause an increased tendency to fall. In this context, age also leads to an overestimation of the personal mobility [19]. Thus, the aforementioned surplus of women in the older age decades again plays an important role [14]. It has also been postulated that women have naturally lower muscle mass, making them more frail in old age [20] and thus more susceptible to falls.

In the *first lockdown*, we found that only female patients suffered sports-related hand injuries. This suggests that women were more involved in individual sports, which were permitted during the lockdown, whereas sports in clubs—where men are most likely to be injured—were prohibited by the government. This is also confirmed by the club statistics of the German Olympic Sports Confederation from 2020, which showed that 60% men versus 40% women are active in sports clubs with team sports [21]. Thus, prevention work to avoid hand injuries is extremely important not only for sports clubs but also for individual sports.

The statistics also showed that the occupational accidents of the male patient increased proportionally during the *first lockdown*. Despite increased home office employment, system-relevant occupations, including skilled trades, were allowed to continue [22]. As the leisure activities of the population were reduced, the percentage of occupational accidents increased (Fig 3). As hand and forearm injuries are the most frequent cause of occupational accidents (41%) and thus cause most new accident pensions [2], the best possible hand surgical care to restore the ability to work is of high socio-economic importance. Hence, hand surgery care must be guaranteed at all times, even during lockdown periods. Nevertheless, this study showed that the capacity of hand surgery can be reduced in times when the mobility of society is limited.

### Distribution of age in the course of the pandemic

An essential parameter for developing prevention measures for hand injuries is the age of the patients. Our study showed that female patients aged 61 and older had a high incidence of falls from standing height, often resulting in a distal radius fracture. An analysis of the Swedish Fracture Registry showed that elderly women suffer distal radius fractures more often than elderly men [23]. Female patients are mostly injured during everyday activities which are not affected by any lockdown. Hung et al. found that the risk of fracture in low-energy trauma increases with lower bone density [24]. Thus, our study also suggested that significantly more older women than men suffer a distal radius fracture due to low-energy trauma resulting from a fall from standing height, which is due to the propensity for osteoporosis after menopause [13]. It can also be concluded that there were proportionally fewer high-energy traumas during lockdown periods.

In addition to older female patients, young men were the second group to suffer accidents. There is evidence that more boys than girls suffer forearm fractures in percentage terms [25], and that young men are more willing to take risks and have a higher mortality rate [26], which was supported by the results of our study.

### Hand trauma rates versus mobility of the population

Most injuries to the distal forearm occur from low-energy trauma and falls from standing height [27], as confirmed by the present study. Ryan et al. described that children also suffer hand injuries primarily from a fall event [25]. However, no study showed a correlation between hand injuries and mobility behaviour in the general population. Our study showed that there is a direct relationship between the mobility behaviour of the population and hand accidents (Fig 5B). In addition, the elective case numbers also showed that they correlate with the mobility data of the population equivalently to the hand trauma case numbers (Figs 5B and 6A). With this knowledge, the hand surgery care centres can better plan surgical capacities and the corresponding staffing ratio in advance.

### First Covid-19 wave versus second Covid-19 wave

The changes in emergency care during the Covid-19 pandemic have been analysed in a number of studies. Pikoulis et al. investigated the epidemiological changes in emergency department in Greece during the *first* and *second lockdowns* in general. Overall, there was a decrease in the number of cases, but during the lockdown phases there was a dynamic change in the spectrum of diagnosis at the emergency department [28]. A study from New Delhi examined the changes in the field of traumatology and orthopaedics over the pandemic year 2020. It showed a reduction in the number of cases both in the field of acute traumatological care and in the field of elective orthopaedics [29]. However, there is a lack of more in-depth information on the cause and progress of the decline in patient numbers.

The present study showed that the activity of the population changed steadily with the duration and accumulated experience of the pandemic. While during the first wave the population had more or less complied to the curfew of the *first lockdown*, the second Covid-19 wave showed a self-responsible mobility behaviour of the population. The *first lockdown* was characterised by a significant decrease in the average number of traumatological operations compared to the previous period (Fig 5C). During the *second lockdown*, a similar daily number of traumatology patients were operated on as in the *pre-pandemic phase* and the *intermediate phase*. This showed that a state of emergency in society, such as a lockdown, only has a short-term impact on hand surgery case numbers and that hand surgery care must be guaranteed in the hospital at all times. In the case of elective patients, there was a significant reduction in operations during the *first* and *second lockdown* compared to the previous period. It is noteworthy that elective operations were also performed in individual cases during the lockdowns. Therefore, the local infection situation was carefully weighed against the disadvantages of postponement for the patient (Fig 6B).

### Limitations

In our study, only surgically treated patients who were treated in the department of hand surgery were examined. In consequence, local regulations may affect the pool of patients treated. Furthermore, despite some socioeconomic relevance, conservatively treated patients were not included into this study, as these patients are not logged in the HTR DGH.

### Conclusion

The patients of hand traumatology mainly comprise young male and the elderly female patients. Occupational injuries and sporting accidents are an important component of hand injuries in young patients. Low-energy trauma is a problem in older patients. With this knowledge, preventive measures can be developed to reduce hand injuries so that young people stay in the labour market and older people do not lose their independence due to a hand injury.

Furthermore, our study showed a correlation between hand traumatology and the mobility behaviour of the population. Thus, it seems possible to predict the number of cases to be expected in hand traumatology in the near future on the basis of the mobility change of the population and to make it usable for capacity planning of hospitals.

The activity behaviour of the population changed steadily with the duration and the experience gained during the pandemic. While in the first wave the population roughly complied with the curfew of the *first lockdown*, the second Covid-19 wave showed a self-responsible adjustment of mobility behaviour, independent of the governments`lockdown measures. The number of elective surgeries also underwent a dynamic during the Covid-19 pandemic. A significant reduction in the number of elective surgeries was recorded during the *first* and *second lockdown*. Considering both patient groups, the hand trauma patients and the elective patients, surgical capacity must be available at all times in a hand surgery centre and be able to be expanded if necessary.
